# Supplementary material for: In Vivo Reprogramming Ameliorates Aging Features in Dentate Gyrus Cells and Improves Memory in Mice
Source: Stem Cell Reports. 2020 Oct 22;15(5):1056–66. doi: 10.1016/j.stemcr.2020.09.010 (PMC7663782; doi:10.1016/j.stemcr.2020.09.010)
Supplement: Document S1. Supplemental Experimental Procedures and Figures S1–S4 [file mmc1.pdf]

Stem Cell Reports, Volume *15*

Supplemental Information

In Vivo Reprogramming Ameliorates Aging Features in Dentate Gyrus Cells and Improves Memory in Mice

Alberto Rodríguez-Matellán, Noelia Alcazar, Félix Hernández, Manuel Serrano, and Jesús Ávila

Supplemental Information

In vivo reprogramming ameliorates aging features in dentate gyrus cells and improves memory in mice

Alberto Rodríguez-Matellán [1,2], Noelia Alcazar [3,4], Félix Hernández [1,2], Manuel Serrano [3,4*] & Jesús Avila [1,2*]

[1] Department of Molecular Neuropathology, Centro de Biología Molecular Severo Ochoa, CBMSO, CSIC-UAM, Madrid, Spain

[2] Center for Networked Biomedical Research on Neurodegenerative Diseases (CIBERNED), Madrid, Spain.

[3] Institute for Research in Biomedicine (IRB Barcelona), The Barcelona Institute of Science and Technology (BIST), Barcelona, Spain.

[4] Catalan Institution for Research and Advanced Studies (ICREA), Barcelona, Spain.

*Correspondence: javila@cbm.csic.es; manuel.serrano@irbbarcelona.org

Supplementary Figures:

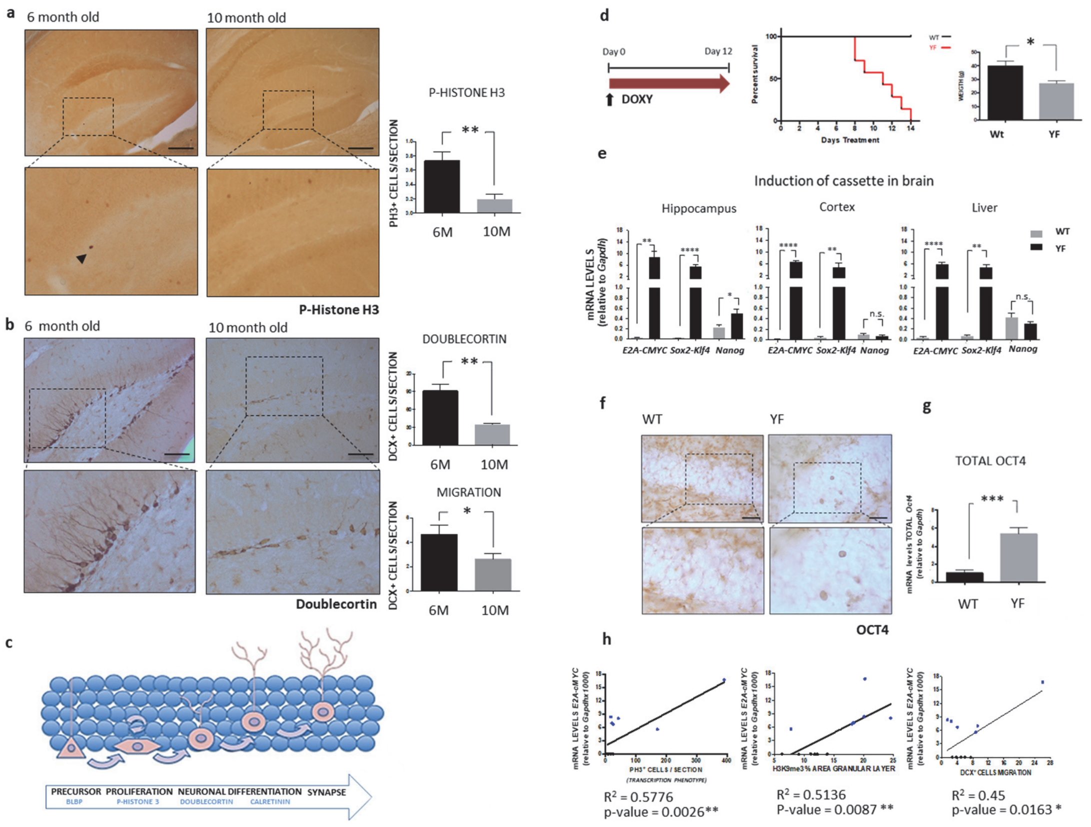

Figure S1. (a) Changes in the levels of phospho-Histone H3 (Ser10) "condensation type" (black arrow) in the dentate gyrus of 6- and 10-month-old wild-type mice. (b) DCX expression in the dentate gyrus of 6- and 10-month-old wild-type mice. For quantifications, protein levels were obtained determining the number of positive cells per section. (c) Scheme of adult neurogenesis in the DG, indicating several neurogenetic markers: BLBP, PH-3 (phosphohistone H3), DCX (doublecortin), and CR (calretinin). Also, there is a final step indicating the integration of newborn neurons into the neuronal network. **Features of the expression of Yamanaka factors following Continuous Protocol.** (d) Schematic diagram of the experimental doxycycline (2 mg/ml, drinking water) administration is shown. Viability (n=10 wild-type and n=7 YFs-expressing mice) as percent of survival mice during treatment is shown. The weight of control (n=5) and YFs-expressing mice (n=5) is shown as mean ± SEM; *p<0.05. (e) YFs expression in the hippocampus, cortex and liver, as determined by RT/PCR, of transgenic RNAs *E2A-CMYC* and *Sox2-Klf4* in wild-type (grey bars) and Yamanaka mice following Continuous Protocol (black bars). *Nanog* mRNA levels, a trigger of the reprogramming process required for the acquisition of pluripotency, is shown as well. (f) Representative image of changes in the OCT4 levels and their corresponding high-power magnifications in wild-type and YFs-expressing mice. (g) OCT4 expression in the hippocampus as determined by RT/PCR. Mean ± SEM. (h) Correlation between YFs expression (measured as *mRNA-E2A-CMYC* levels) and number of phospho-Histone H3 (Ser10) "transcription type" positive-cells, H3K9me3 levels and DCX+-migrating cells. Black dots: wild-type samples; blue dots: YFs-expressing mice samples. Coefficients of determination (R2) and *p* values are shown. Mean ± SEM; *p<0.05, **p< 0. 01, ****p<0.0001, Student's t-test. Bars=200 µm in (a), 100 µm (b), 50 µm in (f).

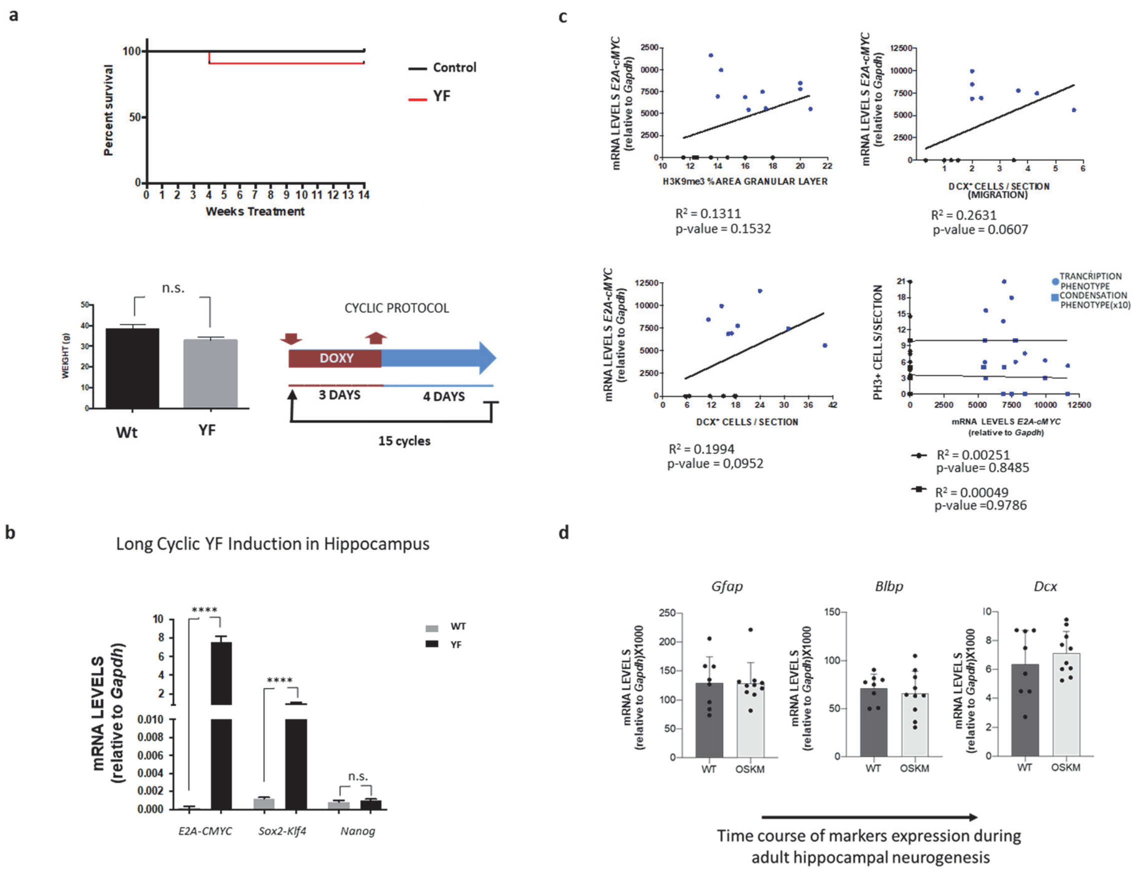

Figure S2. Features of the expression of Yamanaka factors in the hippocampus following cycled protocol . (a) Length of doxycycline treatment (2 mg/ml, drinking water), and viability (n=9 wild-type and n=11 YFs-expressing mice) as percent survival mice after treatment. The weight of control (n=15) and YFs-expressing mice (n=10) is shown as mean ± SEM; n.s.=non statistically significant. Schematic diagram of the experimental doxycycline administration is shown. (b) Hippocampal YFs expression, as determined by RT/PCR, of transgenic *RNA E2A-CMYC* and *Sox2-Klf4* in wild-type (grey bars) and Yamanaka mice following cycled protocol (black bars). *Nanog* mRNA levels is shown as well. ****p< 0. 0001, Student's t- test. (c) Correlation between YFs expression (measured as *mRNA-E2A-CMYC levels*) and H3K9me3 levels, or total DCX+ cells or DCX+ migratory cells. Phosphohistone H3 (Ser10) "transcription type" (circle) and the "condensation type" (square) positive-cells is also shown, black dots: wild-type samples; blue dots: YFs-expressing mice samples. Linear regression analyzes were performed on all mice simultaneously, and their coefficients of determination (R2), *p* values and regression lines (graphic representation) are shown. (d) mRNA levels of the indicated genes in the hippocampus of wild-type (WT) and Yamanaka (OSKM) mice following cycled protocol . Values correspond to the average ± SD of 8 or 10 mice per group (*n*=8 or 10). Statistical significance to wt mice was assessed using unpaired Student's t-test with Welch's correction.

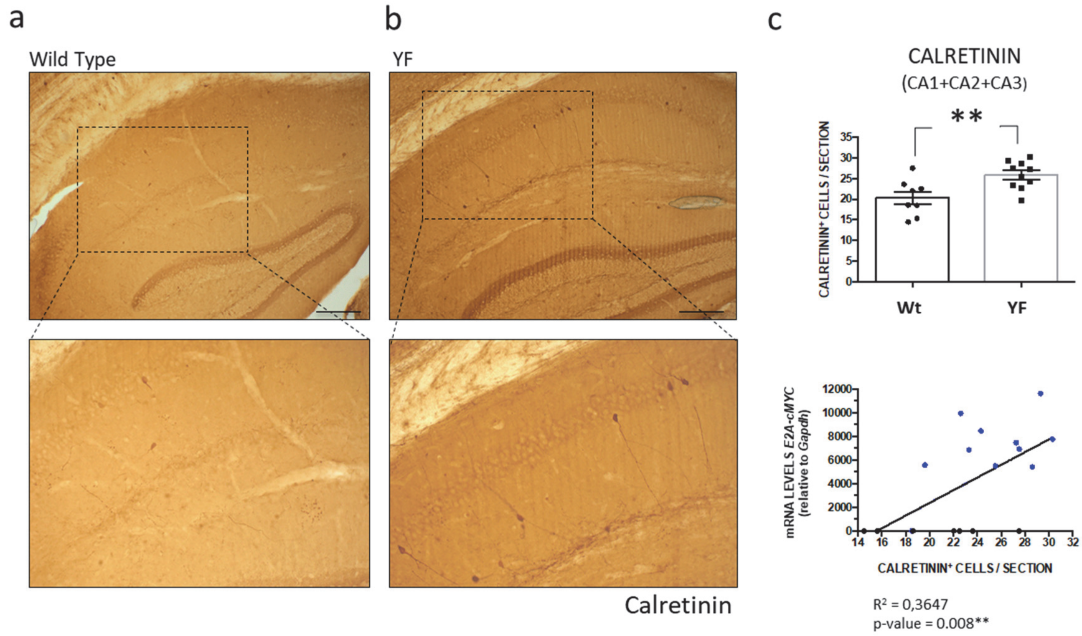

Figure S3. Effect of the cyclic induction of Yamanaka factors on calretinin+ cells outside the granular cell layer. Representative images of calretinin-positive cells and their corresponding high-power magnifications in (a) wild-type mice and (b) YFs-expressing mice. (c) For quantifications, number of Calretinin+ cells present in CA1+CA2+CA3 were obtained determining the number of cells per section. Mean ± SEM; **p < 0.01, Student's t-test). There was a statistical positive correlation between number of Caretinin+ cells and YFs expression (measured as *mRNA-E2A-CMYC* levels). Black dots: wild-type samples; blue dots: YFs-expressing mice samples. Bars=200 µm.

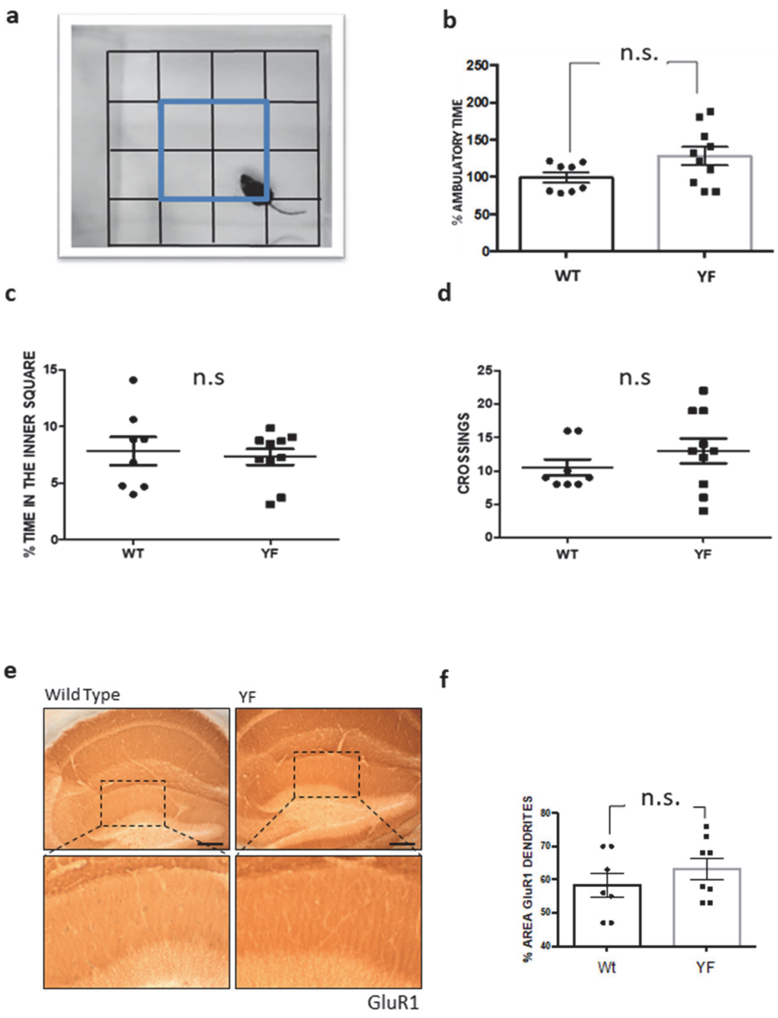

Figure S4. Effect of the expression of Yamanaka factors on parameters related to locomotion and anxiety using the open field test. (a) On the left a representative image of a mouse subjected to test. (b**)** To evaluate if the expression of YFs could modify some aspects related to mobility, the ambulatory time was carried out for wt and YFs-expressing mice. The ambulatory time represents the time in motion that the mice spend in the box for 10 minutes. The data is shown as a percentage of the average time spent, taking 100% the time spent by wt mice. No differences between WT and YFs-expressing mice were found although a non-significant increase was observed (p =0.084). (c) To analyze the anxiety, the time that the WT and YFs-expressing mice enters in the inner square was registered. The data is shown as the percentage of the time the mice spend in the inner square. Crossings are also shown (d). No differences were observed in time spent in the inner square and crossings between the periphery and the center. Mean ± SEM; n.s.= non-statistically significant. **Effect of the cyclic induction of Yamanaka factors on GluR1 staining cells outside the granular layer.** (e) Representative images of GluR1-positive cells and their corresponding high-power magnifications in wild-type mice and YFs-expressing mice. (f) For quantifications, levels in the molecular layer. Dots: wild-type samples; Squares: YFs-expressing mice samples. Mean ± SEM; n.s.= non-statistically significant. Bars=200 µm.

Supplementary Experimental Procedure:

Open field test. Mice aged 10 months were used for this test (9–10 mice per group). The test was performed essentially as described previously (Hall, 1934; Seibenhener and Wooten, 2015), with some modifications. Firstly, the mice were deposited for 10 minutes on the corner of the 45x45 cm plastic opaque box with vertical walls and a grid drawn with sixteen identical squares on the ground, forming its four central squares the inner square. After each exposure, the cage was wiped with 70% ethanol to eliminate odors. Then, some parameters related to anxiety and locomotion were analyzed (ambulatory time, time in the inner square and crossings). For ambulatory time was recorded the time in motion that the mice spent in the box for 10 minutes. The data is shown as a percentage of the average time spent, taking 100% the time spent by WT mice. For the time in the inner square, the percentage of the total time that the mice entered the inner square with three legs was recorded. For the crosses, the times that the mice crossed the box through the inner square were quantified.

Bibliography:

Hall CS. (1934). Emotional behavior in the rat: defecation and urination as measures of individual differences in emotionality, J. Comp. Psychol. *18*, 385–403.

Seibenhener, M.L., and Wooten, M.C. (2015). Use of the Open Field Maze to measure locomotor and anxiety-like behavior in mice. Journal of visualized experiments : JoVE, e52434.
