# Supplementary material for: In Vivo Reprogramming Ameliorates Aging Features in Dentate Gyrus Cells and Improves Memory in Mice
Source: Stem Cell Reports. 2020 Oct 22;15(5):1056–66. doi: 10.1016/j.stemcr.2020.09.010 (PMC7663782; doi:10.1016/j.stemcr.2020.09.010)
Supplement: Document S2. Article plus Supplemental Information [file mmc2.pdf]

Stem Cell Reports
Report
OPEN ACCESS

In Vivo Reprogramming Ameliorates Aging Features in Dentate Gyrus Cells and Improves Memory in Mice

Alberto Rodríguez-Matellán,[1,2] Noelia Alcazar,[3,4] Félix Hernández,[1,2] Manuel Serrano,[3,4,*] and Jesús Ávila[1,2,*]
[1]Department of Molecular Neuropathology, Centro de Biología Molecular Severo Ochoa, CBMSO, CSIC-UAM, Madrid, Spain
[2]Center for Networked Biomedical Research on Neurodegenerative Diseases (CIBERNED), Madrid, Spain
[3]Institute for Research in Biomedicine (IRB Barcelona), The Barcelona Institute of Science and Technology (BIST), Barcelona, Spain
[4]Catalan Institution for Research and Advanced Studies (ICREA), Barcelona, Spain
*Correspondence: manuel.serrano@irbbarcelona.org (M.S.), javila@cbm.csic.es (J.Á.)
https://doi.org/10.1016/j.stemcr.2020.09.010

SUMMARY

Post-translational epigenetic modifications take place in mouse neurons of the dentate gyrus (DG) with age. Here, we report that age-dependent reduction in H3K9 trimethylation (H3K9me3) is prevented by cyclic induction of the Yamanaka factors used for cell reprogramming. Interestingly, Yamanaka factors elevated the levels of migrating cells containing the neurogenic markers doublecortin and calretinin, and the levels of the NMDA receptor subunit GluN2B. These changes could result in an increase in the survival of newborn DG neurons during their maturation and higher synaptic plasticity in mature neurons. Importantly, these cellular changes were accompanied by an improvement in mouse performance in the object recognition test over long time. We conclude that transient cyclic reprogramming *in vivo* in the central nervous system could be an effective strategy to ameliorate aging of the central nervous system and neurodegenerative diseases.

INTRODUCTION

As organisms age, some epigenetic markers are modified (Sen et al., 2016). It has been proposed that the removal of these aging-dependent epigenetic modifications may reverse some features of aging (Benayoun et al., 2015; Liu et al., 2013; Pollina and Brunet, 2011). Temporal expression of Oct4, Sox2, Klf4, and c-Myc (also known as the Yamanaka factors [YFs]), used for pluripotency cell reprogramming (Takahashi and Yamanaka, 2006), can cause this removal of epigenetic marks and subsequent reversal of aging features (Ocampo et al., 2016; Sarkar et al., 2020). Indeed, this approach has been successfully used to improve age-associated hallmarks in peripheral tissues of mice (Ocampo et al., 2016; Zhang et al., 2020). However, little attention has been given to the therapeutic use of YFs in the central nervous system.

Importantly, YF expression must be tightly regulated, since it can lead to aberrant mitogenic stimulation or apoptosis (Abad et al., 2013). In this study, we addressed age-dependent changes in brain structures susceptible to premature degeneration. It has been postulated that age-related brain decline mirrors developmental maturation and, accordingly, brain structures with a late development may be the first to degenerate (Douaud et al., 2014). This notion was first described as Ribot's law (Guillin, 2004). The dentate gyrus (DG) exemplifies a brain structure that matures after birth and whose functions decline early with age. For example, the DG of 10-month-old mice shows a clear decrease in adult neurogenesis, the process through which functional neurons are generated from adult neural precursors and integrated into existing circuits (Snyder, 2019). In the adult mouse brain, adult neurogenesis occurs at the interface between the DG and hilus, in a region known as the subgranular zone. This type of neurogenesis is involved in learning and memory (Clelland et al., 2009; van Praag et al., 2002) and is impaired during aging (Kempermann et al., 1998; Kuhn et al., 1996).

Here, we examined several markers for adult neurogenesis in mice. We found impaired adult hippocampal neurogenesis as the animals aged, thereby supporting previous observations (Kempermann et al., 1998; Kuhn et al., 1996). Our results indicate that in mature mice, the expression of YFs results in a partial prevention of those aging-associated changes found in the newborn neurons of adult mice. In addition, YFs show an effect on DG mature neurons that could increase synaptic plasticity in old mice. This increase could explain why mice expressing YFs outperformed same-age wild-type counterparts in a memory test.

RESULTS

Histone-3 Trimethylation at Lysine 9 as a Marker for Aging at DG

H3K9me3 declines with aging (Ocampo et al., 2016; Sarkar et al., 2020). Indeed, one report indicates that knockdown of methyltransferase (SUV39H1), which modifies H3, leads to a reduction of H3K9me3 and induction of cellular aging (Zhang et al., 2015). To test whether the age-dependent decrease of histone methylation could take place at the hippocampus, we examined the levels of H3K9me3 in cells

 © 2020 The Authors.
This is an open access article under the CC BY license (http://creativecommons.org/licenses/by/4.0/).

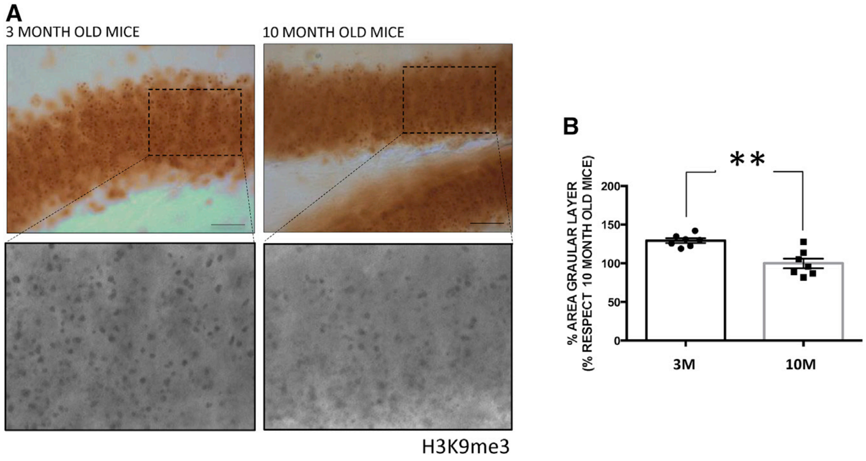

Figure 1. H3K9me3 Modifications Decrease with Age in Granule Cells in the Dentate Gyrus

(A) Representative images of changes in the H3K9me3 levels in 3- and 10-month-old wild-type mice and their corresponding high-power magnifications. Scale bars, 40 μm.

(B) Levels of H3K9me3 in 3- and 10-month-old wild-type mice. For quantifications, levels of H3K9me3 were obtained determining the percentage of the stained area in samples (see Experimental Procedures). The level in 10-month-old wild-type mice was taken as 100% (mean ± SEM; $^{**}p < 0.01$, Student's t test).

present at the DG of 3- and 10-month-old mice. Figure 1 shows a decrease in the presence of H3K9me3 in the granular cells of DG from 10-month-old mice. Thus, since changes were found at the DG, where adult hippocampal neurogenesis (AHN) occurs, we tested for a possible decrease in the levels of markers of adult neurogenesis related to mouse aging.

Markers Related to Adult Neurogenesis Decrease with Mouse Age

AHN decreases in the mouse DG with age, a decrease starting in middle-aged mice (Snyder, 2019). Comparison of two neurogenesis markers, namely phospho-histone H3 (P-H3 modified at serine 10; PH-3-positive, P-$H3^+$) and doublecortin-positive (DCX^+) cells, in the DG of 6- and 10-month-old mice confirmed the age-related decrease in this process in this region of the brain. The DG underwent an approximately 3-fold reduction in the number of P-$H3^+$ cells from 6 to 10 months of age (Figure S1A). Similar results were obtained for DCX^+ cells. Thus, a decrease in total number of DCX^+ cells was observed in 10-month-old compared with 6-month-old mice (Figure S1B). This result agrees with previous data (Snyder, 2019). Also, in Figure S1B, it is shown that DCX^+ cells migrate a shorter distance with age. In addition to alterations of some neurogenic markers (Figure S1C), we turned our attention to possible age-related epigenetic modifications.

Effect of the Yamanaka Factors on Adult Hippocampal Neurogenesis

We hypothesized that expression of YFs in the DG could also affect AHN. For this, we subjected mice of 6 months of age to continuous induction of YFs for 12 days (see continuous protocol in Experimental Procedures) (Figure S1D). The expression of the YFs was confirmed by RT-PCR and immunohistochemistry analysis. Different early and late markers for different stages of AHN (see scheme of Figure S1C) were analyzed. No differences were found in the absence or presence of YF treatment for brain lipid-binding protein-positive ($BLBP^+$) cells, a very early AHN marker (Figure 2A). The immunohistochemistry analysis using P-H3 showed the presence of two types of staining, one darker than the other (Figure 2B). These staining types have been reported previously in hippocampal neurons (Crosio et al., 2003) and other cells (Cheung et al., 2000). The darker staining (condensation) (see black arrow in Figure 2B) indicates an increase in chromosome condensation taking place in mitosis and may point to a higher degree of cell proliferation (Crosio et al., 2003; Goto et al., 1999; Koshland and Strunnikov, 1996). In contrast, the lighter staining may be related to gene expression (transcription) (Labrador and Corces, 2003) at interphase (Chadee et al., 1999; Cheung et al., 2000; Mahadevan et al., 1991) (see white arrow in Figure 2B). No differences in the level of P-H3 related to the "chromatin condensation type" (mitosis) were observed. However, we detected a high variability on the effect of YFs on the level of "chromatin transcription type" that correlates with the high variability found for the expression YFs, although no statistically significant differences were found. Given that an increase, in some cases, takes place in histone phosphorylation and may be related to apoptosis and cell death (Cheung et al., 2000), we studied the effects of YFs expression on cell death (caspase-3 staining). Again, no statistically significant differences were found in response to YF expression induced by this protocol (Figure 2C; see also Figure S1E).

No change in the proportion of DCX^+ cells (AHN late marker) was found. However, these cells showed an increase in migration (Figure 2D), which correlated with YF expression (Figure S1H). It has recently been reported that calcium levels are increased in aged neurons (Pereda et al., 2019). This increase may be related to the decrease of calcium-buffering molecules such as calretinin, which

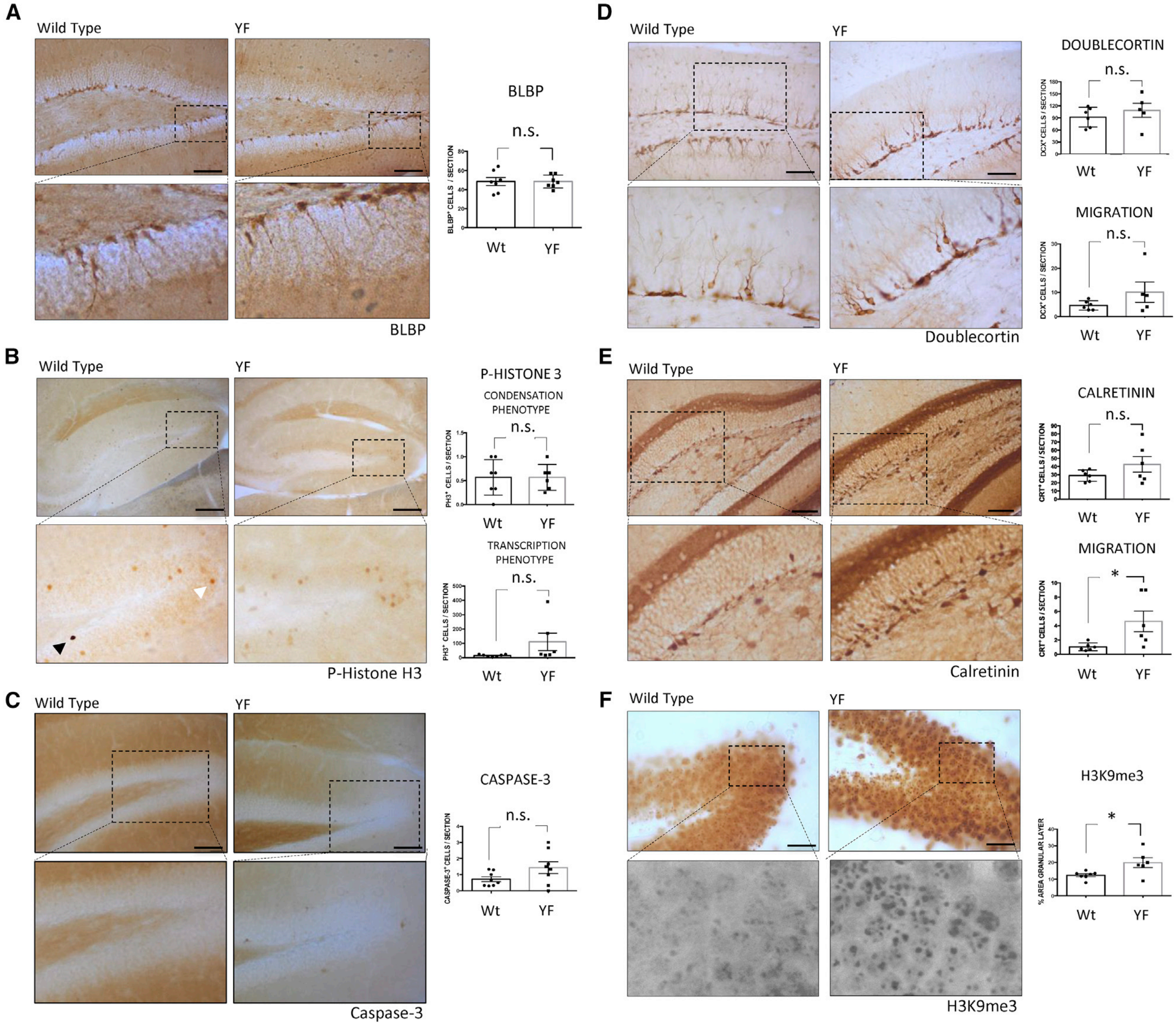

Figure 2. Effect of the Continuous Induction of Yamanaka Factors on the Appearance of Dentate Gyrus Markers for Adult Hippocampal Neurogenesis

We tested the effect of continuous induction of Yamanaka factors (YFs) for 12 days on 6-month-old mice following the continuous protocol (see Experimental Procedures). DG representative images and their corresponding high-power magnifications of (A) number of $BLBP^+$ cells, (B) phospho-histone H3 (PH-3) (Ser10) "transcription type" (white arrow) and "condensation type" (black arrow), (C) number of caspase-3^+ cells, (D) total number and migratory DCX^+ cells, (E) number and migration of $calretinin^+$ cells, and (F) H3K9me3 levels. For quantifications, protein levels in wild-type and YF-expressing mice were obtained determining the number of positive cells localized in the whole DG per slice (A–E) or the percentage of stained area (F). Mean ± SEM; *$p < 0.05$, Student's t test; n.s., not statistically significant. Scale bars, 80 μm (A), 200 μm (B), 100 μm (C–E), and 40 μm (F).

occurs during the aging of the rodent brain (Villa et al., 1994). Thus, we determined the proportion of calretinin-positive ($calretinin^+$) cells in the absence or presence of YFs. We found that the migration of $calretinin^+$ cells is increased in the presence of YFs (Figure 2E). In addition, we observed an increase in the degree of H3K9me3 upon induction of YFs (Figure 2F).

Effect of Cycled Expression of YFs on the Appearance of DG Markers of Adult Hippocampal Neurogenesis

Since the continuous expression of YFs (continuous protocol) led to premature death (Figure S1D), we tested a cyclic protocol (see Experimental Procedures) (Ocampo et al., 2016; Sarkar et al., 2020). Also, we focused on the analysis of mice at 10 months of age, when the levels of AHN are

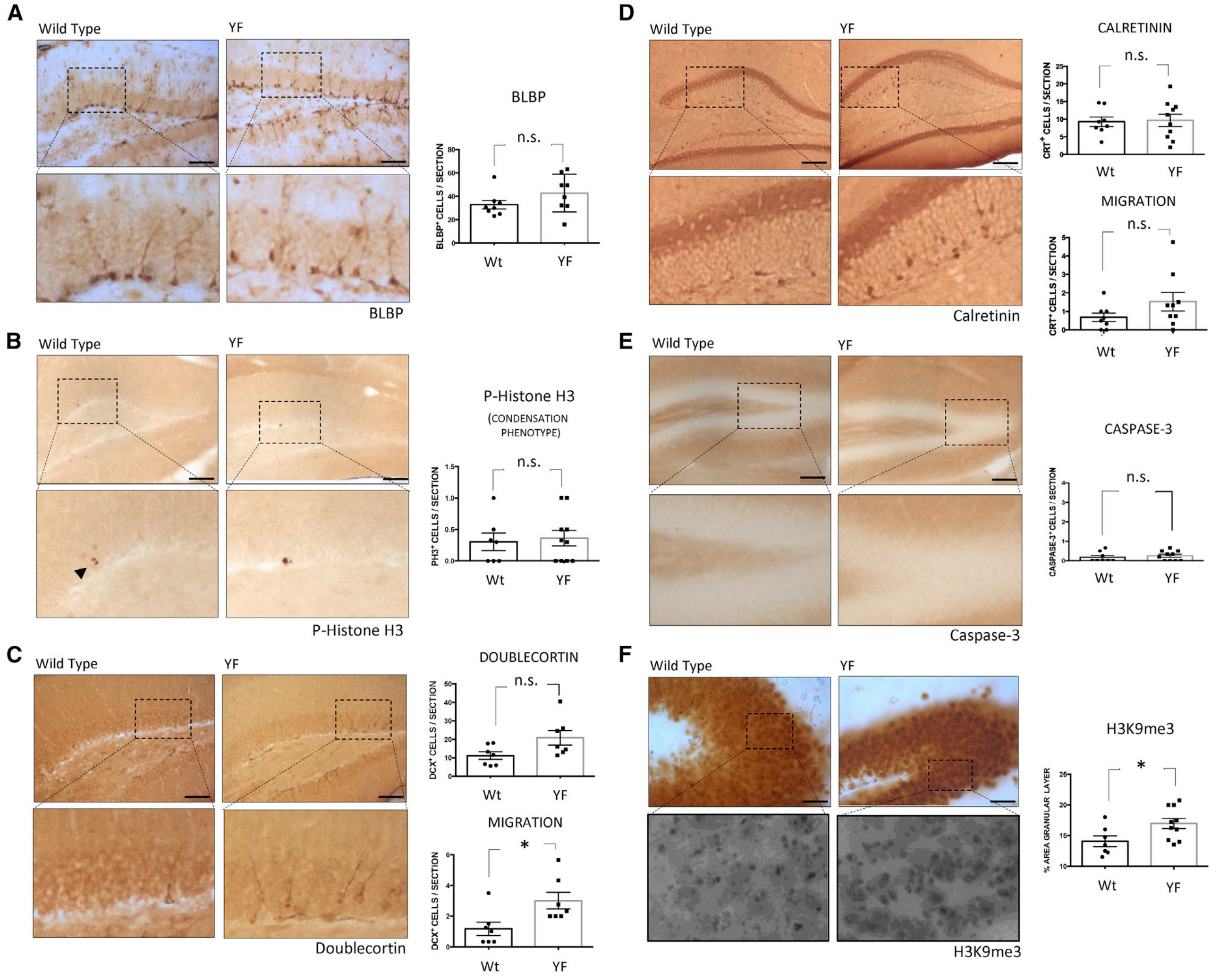

Figure 3. Effect of the Cyclic Induction of Yamanaka Factors on the Appearance of Dentate Gyrus Markers for Adult Hippocampal Neurogenesis

The effect of the cyclic induction of YFs (3 days of doxycycline administration, followed by 4 days of doxycycline withdrawal), during 15 weeks (cycled protocol). DG representative images and their corresponding high-power magnifications of (A) number of $BLBP^+$ cells, (B) phospho-histone H3 (Ser10) ("condensation type;" P-H3), (C) total number and migratory DCX^+ cells, (D) number and migration of calretinin$^+$ cells, (E) number of caspase-3$^+$ cells, and (F) H3K9me3 levels. For quantifications, protein levels in wild-type and YF-expressing mice were obtained determining the number of positive cells localized in the whole DG per slice (A–E) or the percentage of stained area (F). Mean ± SEM; *$p < 0.05$, Student's t test; n.s., not statistically significant. Scale bars, 200 μm (A), 100 μm (C–E), 40 μm (B), and 50 μm (F).

substantially lower than at 6 months of age. The expression of YFs and the features of the mice subjected to the cyclic protocol are indicated in Figures S2A–S2C. Mice of 6 months of age were subjected to weekly cycles of 3 days of doxycycline (2 mg/mL) followed by 4 days of doxycycline withdrawal until 10 months of age. Different markers for different stages of AHN (see scheme of Figure S1C) were analyzed. No differences were found in the absence or presence of YF treatment for $BLBP^+$ cells, a very early AHN marker (Figure 3A). We detected no differences in "condensation staining" and a negligible presence of "transcription staining" of phosphorylated H3 upon YF expression (Figure 3B). Also, in DG we tested for DCX^+ cells and found a greater migration of these cells, similar to that found in younger mice (see Figures 3C and S1B). Finally, the number of DCX+ and calretinin+ cells was not affected, neither the migration of calretinin$^+$ cells Figures 3C and 3D. Thus, cyclic induction revealed that the expression of YFs does not alter the number of neurogenic progenitors in the DG. When mRNA levels were analyzed in preparations of the hippocampus, no differences were observed in the expression of markers for neural precursors such as *Gfap*, *Blbp*, or *Dcx*

(Figure S2D). Also, no significant cell death was found under YF expression induced by this protocol (Figure 3E). We conclude that the cyclic induction of the YFs increases the migration of neurogenic progenitors.

Effect of YFs on DG Mature Neurons

An increase in H3K9me3 was observed (Figure 3F) in DG mature cells. Of note, outside the DG of YF-expressing mice, there was an increase in calretinin$^+$ cells (Figure S3). No such increase occurred in other brain regions such as the cortex (not shown). It has been reported that the number of calretinin$^+$ cells tends to decrease in aged rodents (Ahn et al., 2017). Another study proposed the loss of calretinin$^+$ cells in the rodent hippocampus during aging (Villa et al., 1994). Thus, our results shown in Figure S3 are consistent with these previous reports and indicate that the expression of YFs can revert (or prevent) the decrease in the number of calretinin$^+$ cells outside the DG during aging. The increase in calretinin$^+$ cells outside the DG could also suggest an increased expression of synaptic plasticity-related proteins in interneurons present at the DG.

The aforementioned data could also be explained by YF-induced enhancement of proteins whose expression decreases with age. One of these proteins is the GluN2B subunit, which is present in the N-methyl-D-aspartic acid (NMDA) receptor. This protein facilitates calcium permeability and increased synaptic potentiation (Tang et al., 1999; Yashiro and Philpot, 2008), and its levels decrease with age (Paoletti et al., 2013). The proportion of GluN2B-positive neurons in YF-expressing mice was increased compared with wild-type mice, particularly at dendrites (Figure 4D). These observations would explain the better performance of these animals in the behavioral test (see below). As previously indicated, GluN2B is present mainly in young animals (Paoletti et al., 2013). However, the observed increase of GluN2B may reflect changes in neuronal plasticity in already existing neurons. As a control, the level of another synaptic protein, GluR1, was determined in the absence or presence of YF by immunohistochemistry analysis. Figures S4E and S4F show no differences for this synaptic marker when YFs are expressed.

Partial Improvement in the Object Recognition Test for Mice Expressing YFs in the DG

The cyclic expression of YFs appeared to favor, partially, the differentiation of new neurons in the DG (Figure 3). The last step in AHN is the integration of newborn neurons into the existing hippocampal circuitry (Gonçalves et al., 2016), a process related to the formation of new memories (Deng et al., 2010). However, as previously indicated, behavioral changes could be also due to an increase in synaptic plasticity that may occur in YF-reprogrammed DG mature neurons. Indeed, these changes in mature neurons may be more relevant than those described for AHN in possible behavior tests; thus, we conducted several tests. In this regard, we observed no differences in the behavior of YF-expressing mice in ambulatory time (related to mobility) compared with wild-type controls (Figures S4A and S4B). Also, no changes were found in the presence or absence of YFs for the anxiety measured in the open field test (Figures S4C and S4D). Thus we focused on a memory-related test, namely the object recognition test. We performed this test at two time points in wild-type mice and in mice expressing YFs. No significant differences in performance between the two groups of mice were detected at 2 h after object familiarization. In both cases, the animals showed a similar learning capacity (Figure 4A). However, significant differences were found at 5 days post familiarization. In this regard, wild-type mice were unable to recognize the new object while YF-expressing counterparts did (Figure 4B). In the Memory Index (MI) test, YF-expressing mice outperformed wild-type mice. Moreover, we found a correlation between an increased MI and the degree of induced YF expression (Figure 4C). This increase in MI could be related to the previously mentioned increase of the GluN2B subunit of the NMDA receptor found in YF-treated mice (Figure 4D).

DISCUSSION

Aging is the main risk factor for neurodegenerative disorders. At the DG, aging may result in a decrease of AHN or may affect epigenetic changes in mature granule cells. The presence of YFs may reprogram the function of newborn neurons generated during AHN or that of DG mature neurons. We now discuss these two possibilities.

First, adult neurogenesis is a process that requires the proliferation and differentiation of neuronal precursors at the DG (Kempermann et al., 2015). BLBP is a precursor cell marker that did not change in the presence or absence of YFs. Epigenetic modification, by phosphorylation, at H3 (P-H3 at serine 10) (Cheung et al., 2000) has been used as a cell proliferation marker in AHN (Hans and Dimitrov, 2001; Kim et al., 2017) in the hippocampus, as the modification is related to the number of mitotic cells. However, although age-dependent changes were found in P-H3 (see also Snyder, 2019), no changes were observed in relation to the expression of YFs. Therefore, the expression of the YFs does not seem to increase the generation of new precursors. Thus, we propose that the expression of YFs does not increase the number of precursors but favors the migration of pre-existent precursors from the subgranular zone and survival of newborn neurons.

Our aim here has been to rejuvenate old hippocampal neurons by expressing YFs. However, an extended expression of

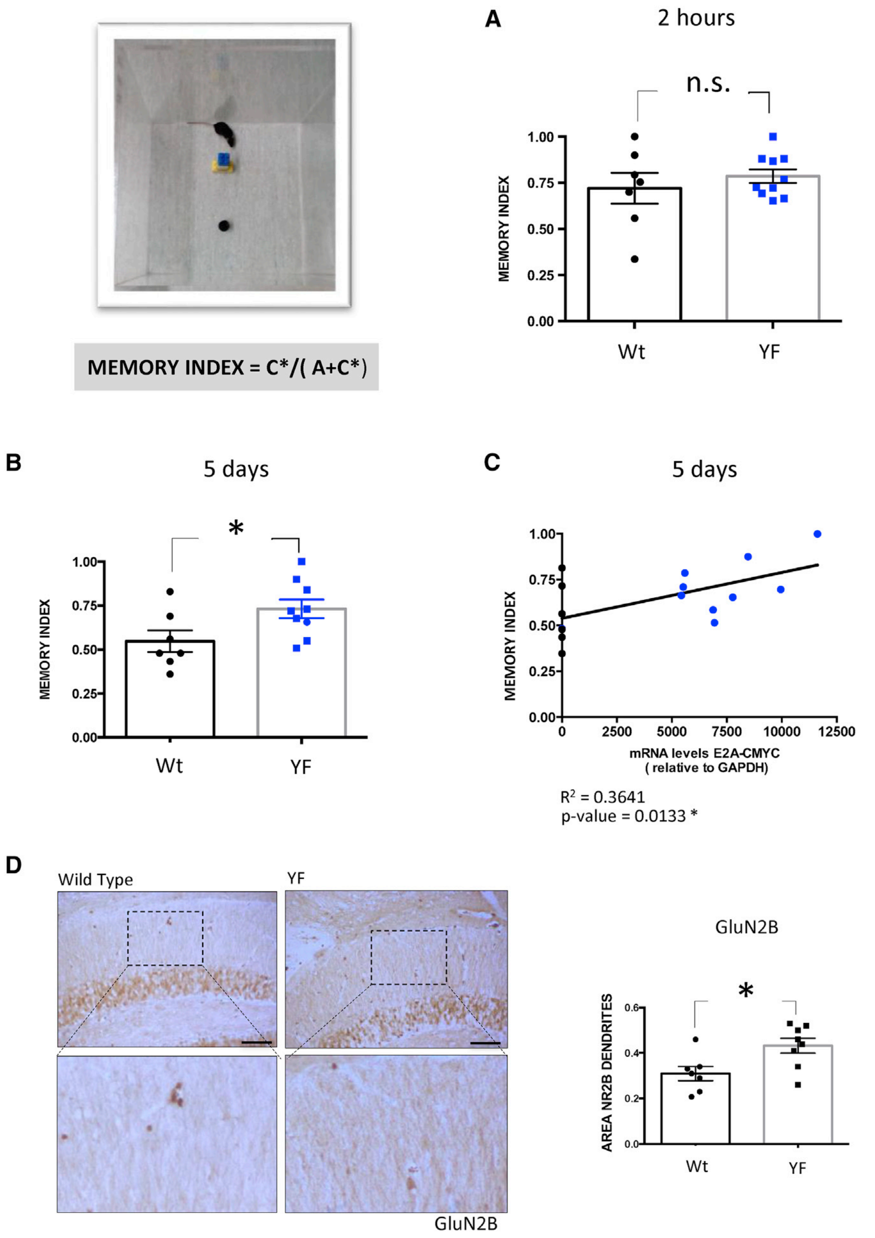

Figure 4. Induction of Yamanaka Factors Improves Performance in the Object Recognition Test, as Measured by Memory Index

(Top left) Representative image of a mouse subjected to object recognition test.

(A and B) (A) The Memory Index was analyzed 2 h after the familiarization trial. Wild-type and YF-expressing mice showed a similar capacity to remember the object previously recognized. (B) After 5 days, wild-type mice had forgotten the objects included in the previous test, but YF-expressing mice showed memory retention.

(C) There is a correlation between YF expression (measured as *mRNA-E2A-CMYC* levels) and improved performance (measured as Memory Index) in the test 5 days after the initial object recognition test.

(D) GluN2B levels in the molecular layer. Black dots, wild-type samples; blue dots, YF-expressing mice samples.

Mean ± SEM; $^{*}p < 0.05$, Student's t test, n.s., not statistically significant.

YFs (continuous protocol) can cause aberrant transcription and cell death (Abad et al., 2013). Indeed, around 50% of YF-expressing mice died after 10 days of this protocol. We then tested cyclic induction of YFs (Ocampo et al., 2016). In this protocol, mouse death was prevented.

Second, in this work we also analyzed the effect of increased age on H3 methylation of DG mature neurons. Epigenetic modifications of chromatin may regulate human aging (Zhang et al., 2015). One such modification could be a change (decrease) in the level of H3K9me3. The effects of this histone methylation may differ depending on the chromatin region where it occurs (Hunter et al., 2012; Snigdha et al., 2016). Here, we found that the expression of YFs results in an increase in H3K9me3 in granule cells in the DG. We looked for a possible reversal of other histone hallmarks like H3K9me3 (Ocampo et al., 2016). In this case, the alterations in H3K9me3 were reversed in 10-month-old mice upon YF expression. The observed increase of H3K9me3 was mainly found in the granular layer of the DG, where the cell bodies of mature neurons are present. Thus, the results may suggest changes in synaptic plasticity. We do not know how YFs may facilitate, upstream, H3K9me3 methylation in neuronal cells, although it may occur in a similar way to that found in peripheral tissues (Ocampo et al., 2016). In mammalian cells,

there are several methyltransferases specific for H3K9 trimethylation, including SUV39H1, G9A, GLP, or SETDB1, among others (Zhang et al., 2020). In addition, SETB1 methyltransferase could also trimethylate H3 at lysine 9 (Zhu et al., 2020). On the other hand, histone H1 (Healton et al., 2020) and non-histone proteins, such as HP1 families (Lehnertz et al., 2003), could regulate such modification. In peripheral tissues, methyltransferase SUV39H1 modifies H3, yielding H3K9me3 (Zhang et al., 2015). It should be also analyzed whether knockdown of SUV39H1 may reduce H3 methylation at the DG. Moreover, in future experiments the downstream consequences of such methylation should be analyzed. In peripheral tissues, H3K9me3 defines constitutive heterochromatin and is critical for the maintenance of genome integrity; indeed it is known as "the epigenetic function of the genome" (Janssen et al., 2018). This epigenetic mark recruits the HP1 family of proteins that shields heterochromatin from access by transcriptional and recombination machineries. In senescent cells, alterations of the heterochromatin patterns lead to the expression of retroviral elements and the formation of double-stranded DNA that activates aging-associated inflammation (inflammaging) (De Cecco et al., 2019). Also, defective H3K9me3 renders heterochromatin accessible to the recombination machinery, which leads to widespread recombination between non-allelic repeats creating duplications, deletions, inversions, and translocations (Janssen et al., 2018). Therefore, restoration of H3K9me3 by YFs in old or senescent cells is thought to reduce inflammaging and genomic instability. Little is known about neuronal cells, although a recent review comments on genome aging in the brain (Lodato and Walsh, 2019).

Going back to AHN, we cannot discard that aged precursors (stem) neuronal cells could have epigenetic age-dependent modifications, at DNA methylation level, in a similar way to the observation made in peripheral tissues (see, e.g., López-Otín et al., 2016).

Precursor (stem) cells undergo epigenetic modifications during aging (Chen and Kerr, 2019) that may result in a slow development of AHN. Indeed, the rate of adult neurogenesis and the speed of morphological maturation of newly generated neurons decline in the aging brain (Trinchero et al., 2019). However, the induction of YF expression and reduction of these epigenetic modifications may lead to a faster progression in AHN.

On the other hand, it has also been proposed that molecular coupling of DNA and histone methylations play a role in silencing genes and retrotransposons (Karimi et al., 2011). In this regard, further work should be done to clarify the whole process (as far as is known for peripheral tissues) in neuronal tissues.

Regarding structural changes, in the presence of YFs we also detected an increase in the subunit GluN2B of NMDA receptor that is expressed mainly in young animals (Tang et al., 1999). The increase in GluN2B, as previously indicated for H3K9me3, may suggest that an improvement in synaptic plasticity could be an effect of YF expression. The increase in GluN2B could correlate with an improved performance in the object recognition test, given some significant differences were found at longer-term measurements when YFs are expressed. Regarding a possible correlation between the level of calretinin and memory impairment, calretinin-deficient mice show an impaired induction of long-term potentiation (Schurmans et al., 1997). This finding is consistent with our observation that an increased number of calretinin$^+$ cells enhances performance in a memory test. This better performance could correlate with both an increase in the proportion of GluN2B present in NMDA receptors located in the dendrites of granule cells and increased calcium buffering due to the increased presence of calretinin in neurons. Thus, although YFs could have some effects on AHN, probably their action could be more relevant in DG mature neurons from old mice by improving synaptic plasticity.

EXPERIMENTAL PROCEDURES

Transgenic Mice

Reprogrammable i4F-B mice were generated as previously described (Abad et al., 2013). In brief, a transgenic mouse line was obtained carrying the transcriptional activator (rtTA) within the ubiquitously expressed *Rosa 26* locus and a single copy of a lentiviral doxycycline-inducible polycistronic cassette encoding the four murine factors Oct4, Sox2, Klf4, and c-Myc (OSKM factors or YFs). The integration site of the transgene is within an intron of the *Pparg* gene. The transgenic line i4F-B contains a functional inducible reprogramming transgene that is expressed in most tissues without affecting the endogenous genes. Heterozygous mice expressing that polycistronic cassette (with a C57BL/6 genetic background) were analyzed and compared with wild-type mice (mice from the same colony that have not inherited the polycistronic cassette).

In Vivo Induction of YFs

YFs were induced in reprogrammable mice of the indicated ages by administration of doxycycline (2 mg/mL) (Sigma) in the drinking water. Two protocols were followed. "Continuous protocol" consisted of the continuous administration to 6-month-old mice of doxycycline for 12 days or until death, whichever happened first (see Figures S1D–S1H). "Cyclic protocol" consisted of weekly cycles of 3 days of doxycycline followed by 4 days of doxycycline withdrawal, starting at 6 months of age, until 10 months age (this protocol had no impact on mortality; see Figure S2).

Animals

Mice were bred in the animal facility at the Centro de Biología Molecular Severo Ochoa, housed in a specific pathogen-free colony facility under standard laboratory conditions following European Community Guidelines (directive 86/609/EEC), and handled in accordance with European and local animal care protocols (PROEX

62/14 and 291/15). They were housed 4–5 per cage with food and water available ad libitum, and maintained in a temperature-controlled environment on a 12/12-h light/dark cycle with light onset at 8 a.m.

Sacrifice and Tissue Processing

Mice were anesthetized with an intraperitoneal pentobarbital injection (Dolethal, 60 mg/kg body weight) and transcardially perfused with saline. Brains were separated into two hemispheres. One hemisphere was removed and fixed in 4% paraformaldehyde in 0.1 M phosphate buffer (PB; pH 7.4) overnight at 4°C. The next day, it was washed three times with 0.1 M PB and cut along the sagittal plane using a vibratome (Leica VT2100S). Serial parasagittal sections (50 μm thick) were cryoprotected in 30% sucrose solution in PB and stored in ethylene glycol/glycerol at −20°C until they were analyzed. The other hemisphere was removed, and the hippocampus was rapidly dissected on ice and frozen in liquid nitrogen for later use in qRT-PCR analysis.

Immunohistochemistry

Before staining, sections were washed with phosphate-buffered saline (PBS) to eliminate the cryoprotective buffer and immersed in 0.3% H_2O_2 in PBS for 30 min to quench endogenous peroxidase activity. Sections were immersed for 1 h in blocking solution (PBS containing 0.5% fetal bovine serum, 0.3% Triton X-100, and 1% bovine serum albumin) and incubated overnight at 4°C with the corresponding primary antibody diluted in blocking solution. After washing, brain sections were incubated first with biotinylated anti-rabbit or anti-mouse secondary antibody and then with avidin-biotin complex using the Elite Vectastain kit (Vector Laboratories, PK-6101-2). Chromogen reactions were performed with diaminobenzidine (SIGMAFASTTM DAB; Sigma, D4293) for 10 min. Hippocampal sections were coverslipped with Fluorosave. Images were captured using an Olympus BX41 microscope with an Olympus Microscope Digital Camera Model DP71 (Olympus Denmark).

Immunohistochemistry Quantifications

Quantifications for P-H3, caspase-3, DCX, and calretinin antibodies were performed by counting the number of positive cells localized in the whole DG (approximately 0.15 mm^2) in four different brain sections. In the case of the quantification of positive calretinin cells, located in the hippocampus but outside of the gyrus dentate, the area taken for quantification was similar to the CA1, CA2, CA3, and subiculum zones (approximately 1.11 mm^2).

For the H3K9me3, GluN2B, and GluR1 antibodies, the quantifications were carried out by measuring the stained area with the ImageJ program. For H3K9me3 antibody, the percentage of area occupied by nuclear foci was measured using a threshold applying the red filter to obtain black-and-white pictures that allow a more precise quantification. For GluN2B and GluR1 antibodies, the whole area occupied by granular cell dendrites were measured using the color deconvolution function of ImageJ and applying the vector H-DAB for this. For measurement of DCX migration, positive cells far from 30 μm (the double of an approximate size of a DCX^+ cell soma) from the subgranular zone were measured.

Quantification of cell migrations was carried out as follows. For study of the localization of DCX^+ or $calretinin^+$ cells, two immunohistochemistry images were taken, per section, using a 40× objective to obtain a composition of the whole DG structure. In the images, the granular layer was divided into zones of 14.5-μm width (a size related to the soma of immature neurons, DCX^+ cells, or $calretinin^+$ cells), being the first zone closest to the subgranular zone where first appear DCX^+ or $calretinin^+$ cells. The cells from that zone were quantified like migrating cells (Fuster-Matanzo et al., 2013).

Antibodies

The following primary antibodies were used: sc-5279 (Santa Cruz Biotechnology) for OCT-3/4; sc-8066 (Santa Cruz Biotechnology) and 326003 (Synaptic Systems) for DCX; ab32423 (Abcam) for BLBP; ab31232 (Abcam) for GluR1; 7697 (Swant) for calretinin; ab8898 (Abcam) for H3K9me3; 07-750 (Millipore) for phospho-histone H3 (Ser10); Y1336 (Phosphosolutions) for GluN2B; and D175 for caspase-3 (Cell Signaling Technology).

Analysis of mRNA Levels

Total RNA was extracted from tissues by means of TRIzol (Invitrogen), following the manufacturer's recommendations. Two micrograms of total RNA was reverse transcribed into cDNA using the iScript Advanced cDNA Synthesis Kit for qRT-PCR (BioRad #172-5038). Real-time qPCR was performed using GoTaq qPCR Master Mix (Promega #A6002) in a 7900HT Fast Real-Time PCR System (Thermo Fisher). Samples were analyzed in triplicate and normalized to *Gapdh*. Calculations were made using the ΔΔCt method. For input normalization, we used the housekeeping gene GAPDH. The primer sequences used were: *E2A-c-Myc* forward GGC TGG AGA TGT TGA GAG CAA, *E2A-c-Myc* reverse AAA GGA AAT CCA GTG GCG C; *Sox2-Klf4* forward ACT GCC CCT GTC GCA CAT, *Sox2-Klf4* reverse CAT GTC AGA CTC GCC AGG TG; *Nanog* forward CAA GGG TCT GCT ACT GAG ATG CTC TG, *Nanog* reverse TTT TGT TTG GGA CTG GTA GAA GAA TCA G; *Oct4* (total) forward GTT GGA GAA GGT GGA ACC AA, *Oct4* (total) reverse CCA AGG TGA TCC TCT TCT GC; m*Dcx* forward AAA CTG GAA ACC GGA GTT GTC, m*Dcx* reverse CGT CTT GGT CGT TAC CTG AGT; m*Gfap* forward CCC TGG CTC GTG TGG ATT T, m*Gfap* reverse GAC CGA TAC CAC TCC TCT GTC; m*Blbp* forward AGA CCC GAG TTC CTC CAG TT, m*Blbp* reverse ATC ACC ACT TTG CCA CCT TC; *Gapdh* forward TTC ACC ACC ATG GAG AAG GC, *Gapdh* reverse CCC TTT TGG CTC CAC CCT.

Object Recognition Task

Mice aged 10 months were used for this test (9–10 mice per group). Mice were tested following cycled protocol (from Monday to Wednesday with doxycycline 2 mg/mL in the drinking water and from Thursday to Sunday with water). The test was performed essentially as described previously (Engel et al., 2006), with a few modifications. In brief, on the first day (Wednesday), the mice were habituated for 10 min in a 45 × 45-cm plastic box with vertical opaque walls. On the second day (Thursday), they were placed in the same box for 5 min, allowing them to explore two identical objects (objects A and B). Both objects were placed on the long axis of the cage, each 13 cm from the cage end. After each exposure, the

objects and the cage were wiped with 70% ethanol to eliminate odors. Two hours after the familiarization trial, each mouse was released into the open field with one of the old objects (object A) and a new one (object C). The position of object C was the same as that of object B in the familiarization trial. The mice were given 5 min to explore the box (Test 1). Five days later (Tuesday), the mice were released again into the open field, with object A in the same position and another new object (object D). The position of the latter was the same as that of object C in Test 1. Again, animals were allowed 5 min to explore the box (Test 2). Animals were considered to show recognition when their head was <2 cm from the object. In Test 1, the time (tA and tC) the animal spent exploring the two objects (objects A and C, respectively) was recorded. The MI, defined as the ratio of time spent exploring the new object to the time spent exploring both objects (MI = [tC/(tA + tC)] × 100), was used to measure non-spatial memory. In Test 2 the same MI was applied, replacing tC with the time spent exploring the object D, tD (MI = [tD/(tA + tD)] × 100).

Statistical Analysis

The data are presented as mean values ± SEM. Statistical analyses of data were performed by applying a Student's t test for each statistical comparison. p values of <0.05 were considered significant. Linear regression analyses were performed on all mice simultaneously, and their coefficients of determination (R^2), p values, and regression lines (graphic representation) are shown in the figures.

SUPPLEMENTAL INFORMATION

Supplemental Information can be found online at https://doi.org/10.1016/j.stemcr.2020.09.010.

AUTHOR CONTRIBUTIONS

A.R.-M. performed most of the experiments and contributed to data analysis, design, and discussion. N.A. performed qPCR analysis and contributed to data analysis. F.H., M.S., and J.A. designed and supervised the study, secured funding, analyzed the data, and wrote the manuscript. All authors discussed the results and commented on the manuscript.

CONFLICTS OF INTEREST

The authors declare that they have no conflict of interest. Correspondence and requests for materials should be addressed to J.A. (javila@cbm.csic.es). and M.S. (manuel.serrano@irbbarcelona.org).

ACKNOWLEDGMENTS

General: The authors would like to thank Raquel Cuadros for her technical contribution and Optical and Confocal Microscopy Service from CBMSO for technical support. N.A. and A.R.-M. were recipients of an FPI contract from the Spanish Ministry of Economy (MINECO).

Funding: Work in the laboratory of J.A. is funded by grants from Spanish Ministry of Economy and Competitiveness (PGC-2018-09177-B-100) and Centro de Investigación Biomédica en Red sobre Enfermedades Neurodegenerativas (CIBERNED, ISCIII). Work in the laboratory of F.H. is funded by grants from Spanish Ministry of Economy and Competitiveness (BFU2016-77885-P) and Comunidad de Madrid co-financed with the Structural Funds of the European Union (S2017/BMD-3700 (NEUROMETAB-CM)). Work in the laboratory of M.S. was funded by the IRB and by grants from the MINECO co-funded by the European Regional Development Fund (SAF2017-82613-R), the European Research Council (ERC-2014-AdG/669622), and "La Caixa" Foundation. An institutional grant from the Fundación Ramón Areces to the CBMSO is also acknowledged.

Received: March 20, 2020
Revised: September 23, 2020
Accepted: September 24, 2020
Published: October 22, 2020

REFERENCES

Abad, M., Mosteiro, L., Pantoja, C., Canamero, M., Rayon, T., Ors, I., Grana, O., Megias, D., Dominguez, O., Martinez, D., et al. (2013). Reprogramming in vivo produces teratomas and iPS cells with totipotency features. Nature *502*, 340–345.

Ahn, J.H., Hong, S., Park, J.H., Kim, I.H., Cho, J.H., Lee, T.K., Lee, J.C., Chen, B.H., Shin, B.N., Bae, E.J., et al. (2017). Immunoreactivities of calbindinD28k, calretinin and parvalbumin in the somatosensory cortex of rodents during normal aging. Mol. Med. Rep. *16*, 7191–7198.

Benayoun, B.A., Pollina, E.A., and Brunet, A. (2015). Epigenetic regulation of ageing: linking environmental inputs to genomic stability. Nat. Rev. Mol. Cell Biol. *16*, 593–610.

Clelland, C.D., Choi, M., Romberg, C., Clemenson, G.D., Jr., Fragniere, A., Tyers, P., Jessberger, S., Saksida, L.M., Barker, R.A., Gage, F.H., et al. (2009). A functional role for adult hippocampal neurogenesis in spatial pattern separation. Science *325*, 210–213.

Crosio, C., Heitz, E., Allis, C.D., Borrelli, E., and Sassone-Corsi, P. (2003). Chromatin remodeling and neuronal response: multiple signaling pathways induce specific histone H3 modifications and early gene expression in hippocampal neurons. J. Cell Sci. *116*, 4905–4914.

Chadee, D.N., Hendzel, M.J., Tylipski, C.P., Allis, C.D., Bazett-Jones, D.P., Wright, J.A., and Davie, J.R. (1999). Increased Ser-10 phosphorylation of histone H3 in mitogen-stimulated and oncogene-transformed mouse fibroblasts. J. Biol. Chem. *274*, 24914–24920.

Chen, D., and Kerr, C. (2019). The epigenetics of stem cell aging comes of age. Trends Cell Biol. *29*, 563–568.

Cheung, P., Allis, C.D., and Sassone-Corsi, P. (2000). Signaling to chromatin through histone modifications. Cell *103*, 263–271.

De Cecco, M., Takahiro Ito, T., Petrashen, A.P., Elias, A.E., Skvir, N.J., Criscione, S.W., Caligiana, A., Brocculi, G., Adney, E.M., Boeke, J.D., et al. (2019). L1 drives IFN in senescent cells and promotes age-associated inflammation. Nature *566*, 73–78.

Deng, W., Aimone, J.B., and Gage, F.H. (2010). New neurons and new memories: how does adult hippocampal neurogenesis affect learning and memory? Nat. Rev. Neurosci. *11*, 339–350.

Douaud, G., Groves, A.R., Tamnes, C.K., Westlye, L.T., Duff, E.P., Engvig, A., Walhovd, K.B., James, A., Gass, A., Monsch, A.U., et al. (2014). A common brain network links development, aging, and vulnerability to disease. Proc. Natl. Acad. Sci. U S A *111*, 17648–17653.

Engel, T., Hernandez, F., Avila, J., and Lucas, J.J. (2006). Full reversal of Alzheimer's disease-like phenotype in a mouse model with conditional overexpression of glycogen synthase kinase-3. J. Neurosci. *26*, 5083–5090.

Fuster-Matanzo, A., Llorens-Martin, M., Sirerol-Piquer, M.S., Garcia-Verdugo, J.M., Avila, J., and Hernandez, F. (2013). Dual effects of increased glycogen synthase kinase-3beta activity on adult neurogenesis. Hum. Mol. Genet. *22*, 1300–1315.

Gonçalves, J.T., Schafer, S.T., and Gage, F.H. (2016). Adult neurogenesis in the Hippocampus: from stem cells to behavior. Cell *167*, 897–914.

Goto, H., Tomono, Y., Ajiro, K., Kosako, H., Fujita, M., Sakurai, M., Okawa, K., Iwamatsu, A., Okigaki, T., Takahashi, T., et al. (1999). Identification of a novel phosphorylation site on histone H3 coupled with mitotic chromosome condensation. J. Biol. Chem. *274*, 25543–25549.

Guillin, V. (2004). Theodule Ribot's ambiguous positivism: philosophical and epistemological strategies in the founding of French scientific psychology. J. Hist. Behav. Sci. *40*, 165–181.

Hans, F., and Dimitrov, S. (2001). Histone H3 phosphorylation and cell division. Oncogene *20*, 3021–3027.

Healton, S.E., Pinto, H.D., Mishra, L.N., Hamilton, G.A., Wheat, J.C., Swist-Rosowska, K., Shukeir, N., Dou, Y., Steidl, U., Jenuwein, T., et al. (2020). H1 linker histones silence repetitive elements by promoting both histone H3K9 methylation and chromatin compaction. Proc. Natl. Acad. Sci. U S A *117*, 14251–14258.

Hunter, R.G., Murakami, G., Dewell, S., Seligsohn, M., Baker, M.E., Datson, N.A., McEwen, B.S., and Pfaff, D.W. (2012). Acute stress and hippocampal histone H3 lysine 9 trimethylation, a retrotransposon silencing response. Proc. Natl. Acad. Sci. U S A *109*, 17657–17662.

Janssen, A., Colmenares, S.U., and Karpen, G.H. (2018). Heterochromatin: guardian of the genome. Annu. Rev. Cell Dev. Biol. *34*, 265–288.

Karimi, M.M., Goyal, P., Maksakova, I.A., Bilenky, M., Leung, D., Tang, J.X., Shinkai, Y., Mager, D.L., Jones, S., Hirst, M., et al. (2011). DNA methylation and SETDB1/H3K9me3 regulate predominantly distinct sets of genes, retroelements, and chimeric transcripts in mESCs. Cell Stem Cell *8*, 676–687.

Kempermann, G., Kuhn, H.G., and Gage, F.H. (1998). Experience-induced neurogenesis in the senescent dentate gyrus. J. Neurosci. *18*, 3206–3212.

Kempermann, G., Song, H., and Gage, F.H. (2015). Neurogenesis in the adult hippocampus. Cold Spring Harb Perspect. Biol. *7*, a018812.

Kim, J.Y., Jeong, H.S., Chung, T., Kim, M., Lee, J.H., Jung, W.H., and Koo, J.S. (2017). The value of phosphohistone H3 as a proliferation marker for evaluating invasive breast cancers: a comparative study with Ki67. Oncotarget *8*, 65064–65076.

Koshland, D., and Strunnikov, A. (1996). Mitotic chromosome condensation. Annu. Rev. Cell Dev. Biol. *12*, 305–333.

Kuhn, H.G., Dickinson-Anson, H., and Gage, F.H. (1996). Neurogenesis in the dentate gyrus of the adult rat: age-related decrease of neuronal progenitor proliferation. J. Neurosci. *16*, 2027–2033.

Labrador, M., and Corces, V.G. (2003). Phosphorylation of histone H3 during transcriptional activation depends on promoter structure. Genes Dev. *17*, 43–48.

Lehnertz, B., Ueda, Y., Derijck, A.A.H.A., Braunschweig, U., Perez-Burgos, L., Kubicek, S., Chen, T., Li, E., Jenuwein, T., and Peters, A.H.F.M. (2003). Suv39h-mediated histone H3 lysine 9 methylation directs DNA methylation to major satellite repeats at pericentric heterochromatin. Curr. Biol. *13*, 1192–1200.

Liu, L., Cheung, T.H., Charville, G.W., Hurgo, B.M., Leavitt, T., Shih, J., Brunet, A., and Rando, T.A. (2013). Chromatin modifications as determinants of muscle stem cell quiescence and chronological aging. Cell Rep. *4*, 189–204.

Lodato, M.A., and Walsh, A.C. (2019). Genome aging: somatic mutation in the brain links age-related decline with disease and nominates pathogenic mechanisms. Hum. Mol. Genet. *28*, R197–R206.

López-Otín, C., Galluzzi, L., Freije, J.M.P., Madeo, F., and Guido Kroemer, G. (2016). Metabolic control of longevity. Cell *166*, 802–821.

Mahadevan, L.C., Willis, A.C., and Barratt, M.J. (1991). Rapid histone H3 phosphorylation in response to growth factors, phorbol esters, okadaic acid, and protein synthesis inhibitors. Cell *65*, 775–783.

Ocampo, A., Reddy, P., Martinez-Redondo, P., Platero-Luengo, A., Hatanaka, F., Hishida, T., Li, M., Lam, D., Kurita, M., Beyret, E., et al. (2016). In vivo amelioration of age-associated hallmarks by partial reprogramming. Cell *167*, 1719–1733.

Paoletti, P., Bellone, C., and Zhou, Q. (2013). NMDA receptor subunit diversity: impact on receptor properties, synaptic plasticity and disease. Nat. Rev. Neurosci. *14*, 383–400.

Pereda, D., Al-Osta, I., Okorocha, A.E., Easton, A., and Hartell, N.A. (2019). Changes in presynaptic calcium signalling accompany age-related deficits in hippocampal LTP and cognitive impairment. Aging Cell, e13008.

Pollina, E.A., and Brunet, A. (2011). Epigenetic regulation of aging stem cells. Oncogene *30*, 3105–3126.

Sarkar, T.J., Quarta, M., Mukherjee, S., Colville, A., Paine, P., Doan, L., Tran, C.M., Chu, C.R., Horvath, S., Qi, L.S., et al. (2020). Transient non-integrative expression of nuclear reprogramming factors promotes multifaceted amelioration of aging in human cells. Nat. Commun. *11*, 1545.

Schurmans, S., Schiffmann, S.N., Gurden, H., Lemaire, M., Lipp, H.P., Schwam, V., Pochet, R., Imperato, A., Bohme, G.A., and Parmentier, M. (1997). Impaired long-term potentiation induction in dentate gyrus of calretinin-deficient mice. Proc. Natl. Acad. Sci. U S A *94*, 10415–10420.

Sen, P., Shah, P.P., Nativio, R., and Berger, S.L. (2016). Epigenetic mechanisms of longevity and aging. Cell *166*, 822–839.

Snigdha, S., Prieto, G.A., Petrosyan, A., Loertscher, B.M., Dieskau, A.P., Overman, L.E., and Cotman, C.W. (2016). H3K9me3

inhibition improves memory, promotes spine formation, and increases BDNF levels in the aged hippocampus. J. Neurosci. *36*, 3611–3622.

Snyder, J.S. (2019). Recalibrating the relevance of adult neurogenesis. Trends Neurosci. *42*, 164–178.

Takahashi, K., and Yamanaka, S. (2006). Induction of pluripotent stem cells from mouse embryonic and adult fibroblast cultures by defined factors. Cell *126*, 663–676.

Tang, Y.P., Shimizu, E., Dube, G.R., Rampon, C., Kerchner, G.A., Zhuo, M., Liu, G., and Tsien, J.Z. (1999). Genetic enhancement of learning and memory in mice. Nature *401*, 63–69.

Trinchero, M.F., Herrero, M., Monzon-Salinas, M.C., and Schinder, A.F. (2019). Experience-dependent structural plasticity of adult-born neurons in the aging Hippocampus. Front. Neurosci. *13*, 739.

van Praag, H., Schinder, A.F., Christie, B.R., Toni, N., Palmer, T.D., and Gage, F.H. (2002). Functional neurogenesis in the adult hippocampus. Nature *415*, 1030–1034.

Villa, A., Podini, P., Panzeri, M.C., Racchetti, G., and Meldolesi, J. (1994). Cytosolic Ca^{2+} binding proteins during rat brain ageing: loss of calbindin and calretinin in the hippocampus, with no change in the cerebellum. Eur. J. Neurosci. *6*, 1491–1499.

Yashiro, K., and Philpot, B.D. (2008). Regulation of NMDA receptor subunit expression and its implications for LTD, LTP, and metaplasticity. Neuropharmacology *55*, 1081–1094.

Zhang, W., Li, J., Suzuki, K., Qu, J., Wang, P., Zhou, J., Liu, X., Ren, R., Xu, X., Ocampo, A., et al. (2015). Aging stem cells. A Werner syndrome stem cell model unveils heterochromatin alterations as a driver of human aging. Science *348*, 1160–1163.

Zhang, W., Qu, J., Liu, G.H., and Belmonte, J.C.I. (2020). The ageing epigenome and its rejuvenation. Nat. Rev. Mol. Cell Biol. *21*, 137–150.

Zhu, Y., Sun, D., Mira Jakovcevski, M., and Jiang, Y. (2020). Epigenetic mechanism of SETDB1 in brain: implications for neuropsychiatric disorders. Transl. Psychiatry *10*, 115.

Stem Cell Reports, Volume *15*

Supplemental Information

In Vivo Reprogramming Ameliorates Aging Features in Dentate Gyrus Cells and Improves Memory in Mice

Alberto Rodríguez-Matellán, Noelia Alcazar, Félix Hernández, Manuel Serrano, and Jesús Ávila

Supplemental Information

In vivo reprogramming ameliorates aging features in dentate gyrus cells and improves memory in mice

Alberto Rodríguez-Matellán [1,2], Noelia Alcazar [3,4], Félix Hernández [1,2], Manuel Serrano [3,4*] & Jesús Avila [1,2*]

[1] Department of Molecular Neuropathology, Centro de Biología Molecular Severo Ochoa, CBMSO, CSIC-UAM, Madrid, Spain

[2] Center for Networked Biomedical Research on Neurodegenerative Diseases (CIBERNED), Madrid, Spain.

[3] Institute for Research in Biomedicine (IRB Barcelona), The Barcelona Institute of Science and Technology (BIST), Barcelona, Spain.

[4] Catalan Institution for Research and Advanced Studies (ICREA), Barcelona, Spain.

*Correspondence: javila@cbm.csic.es; manuel.serrano@irbbarcelona.org

Supplementary Figures:

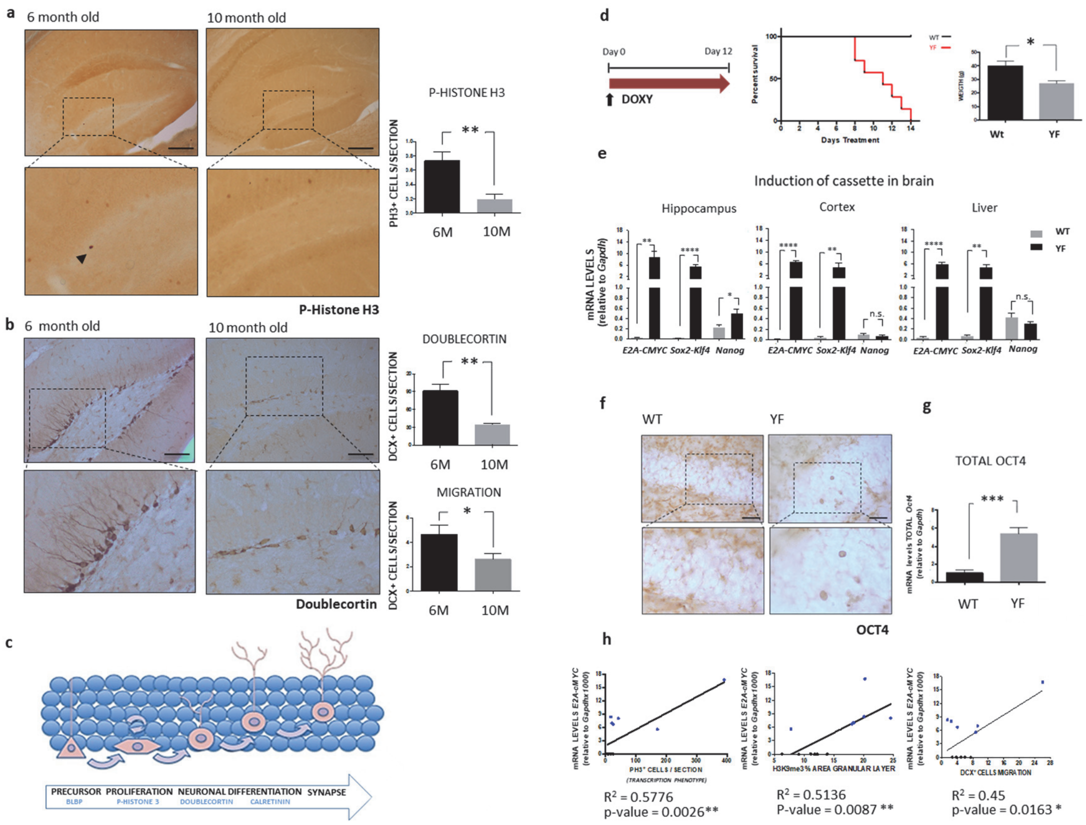

Figure S1. (a) Changes in the levels of phospho-Histone H3 (Ser10) “condensation type” (black arrow) in the dentate gyrus of 6- and 10-month-old wild-type mice. (b) DCX expression in the dentate gyrus of 6- and 10-month-old wild-type mice. For quantifications, protein levels were obtained determining the number of positive cells per section. (c) Scheme of adult neurogenesis in the DG, indicating several neurogenetic markers: BLBP, PH-3 (phosphohistone H3), DCX (doublecortin), and CR (calretinin). Also, there is a final step indicating the integration of newborn neurons into the neuronal network. **Features of the expression of Yamanaka factors following Continuous Protocol.** (d) Schematic diagram of the experimental doxycycline (2 mg/ml, drinking water) administration is shown. Viability (n=10 wild-type and n=7 YFs-expressing mice) as percent of survival mice during treatment is shown. The weight of control (n=5) and YFs-expressing mice (n=5) is shown as mean ± SEM; *p<0.05. (e) YFs expression in the hippocampus, cortex and liver, as determined by RT/PCR, of transgenic RNAs *E2A-CMYC* and *Sox2-Klf4* in wild-type (grey bars) and Yamanaka mice following Continuous Protocol (black bars). *Nanog* mRNA levels, a trigger of the reprogramming process required for the acquisition of pluripotency, is shown as well. (f) Representative image of changes in the OCT4 levels and their corresponding high-power magnifications in wild-type and YFs-expressing mice. (g) OCT4 expression in the hippocampus as determined by RT/PCR. Mean ± SEM. (h) Correlation between YFs expression (measured as *mRNA-E2A-CMYC* levels) and number of phospho-Histone H3 (Ser10) “transcription type” positive-cells, H3K9me3 levels and DCX+-migrating cells. Black dots: wild-type samples; blue dots: YFs-expressing mice samples. Coefficients of determination (R2) and *p* values are shown. Mean ± SEM; *p<0.05, **p< 0. 01, ****p<0.0001, Student's t-test. Bars=200 μm in (a), 100 μm (b), 50 μm in (f).

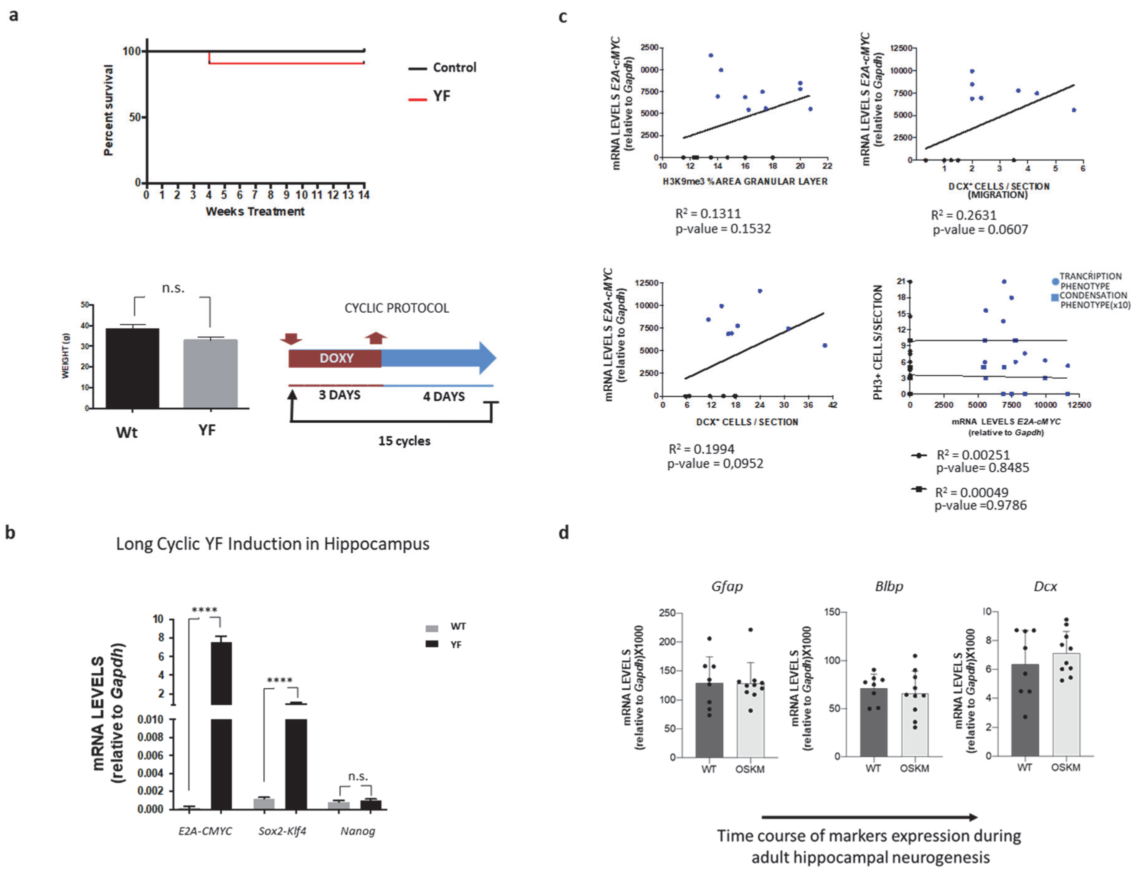

Figure S2. Features of the expression of Yamanaka factors in the hippocampus following cycled protocol . (a) Length of doxycycline treatment (2 mg/ml, drinking water), and viability (n=9 wild-type and n=11 YFs-expressing mice) as percent survival mice after treatment. The weight of control (n=15) and YFs-expressing mice (n=10) is shown as mean ± SEM; n.s.=non statistically significant. Schematic diagram of the experimental doxycycline administration is shown. (b) Hippocampal YFs expression, as determined by RT/PCR, of transgenic *RNA E2A-CMYC* and *Sox2-Klf4* in wild-type (grey bars) and Yamanaka mice following cycled protocol (black bars). *Nanog* mRNA levels is shown as well. ****$p < 0.0001$, Student's t- test. (c) Correlation between YFs expression (measured as *mRNA-E2A-CMYC levels*) and H3K9me3 levels, or total DCX+ cells or DCX+ migratory cells. Phosphohistone H3 (Ser10) "transcription type" (circle) and the "condensation type" (square) positive-cells is also shown, black dots: wild-type samples; blue dots: YFs-expressing mice samples. Linear regression analyzes were performed on all mice simultaneously, and their coefficients of determination (R2), *p* values and regression lines (graphic representation) are shown. (d) mRNA levels of the indicated genes in the hippocampus of wild-type (WT) and Yamanaka (OSKM) mice following cycled protocol . Values correspond to the average ± SD of 8 or 10 mice per group (*n*=8 or 10). Statistical significance to wt mice was assessed using unpaired Student's t-test with Welch's correction.

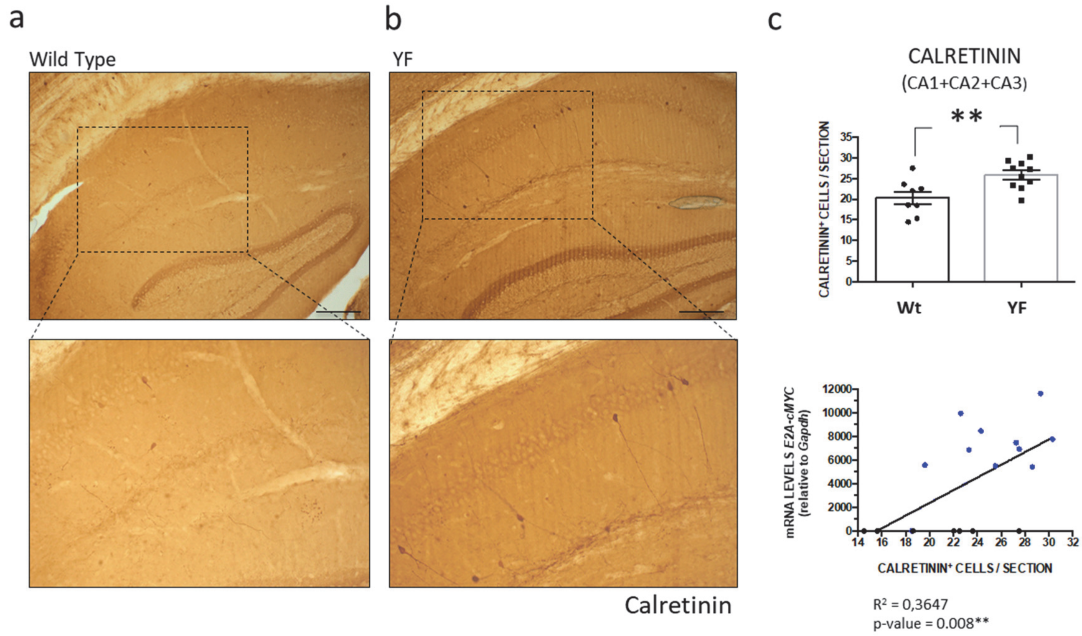

Figure S3. Effect of the cyclic induction of Yamanaka factors on calretinin+ cells outside the granular cell layer. Representative images of calretinin-positive cells and their corresponding high-power magnifications in (a) wild-type mice and (b) YFs-expressing mice. (c) For quantifications, number of Calretinin+ cells present in CA1+CA2+CA3 were obtained determining the number of cells per section. Mean ± SEM; $^{**}p < 0.01$, Student's t-test). There was a statistical positive correlation between number of Caretinin+ cells and YFs expression (measured as *mRNA-E2A-CMYC* levels). Black dots: wild-type samples; blue dots: YFs-expressing mice samples. Bars=200 µm.

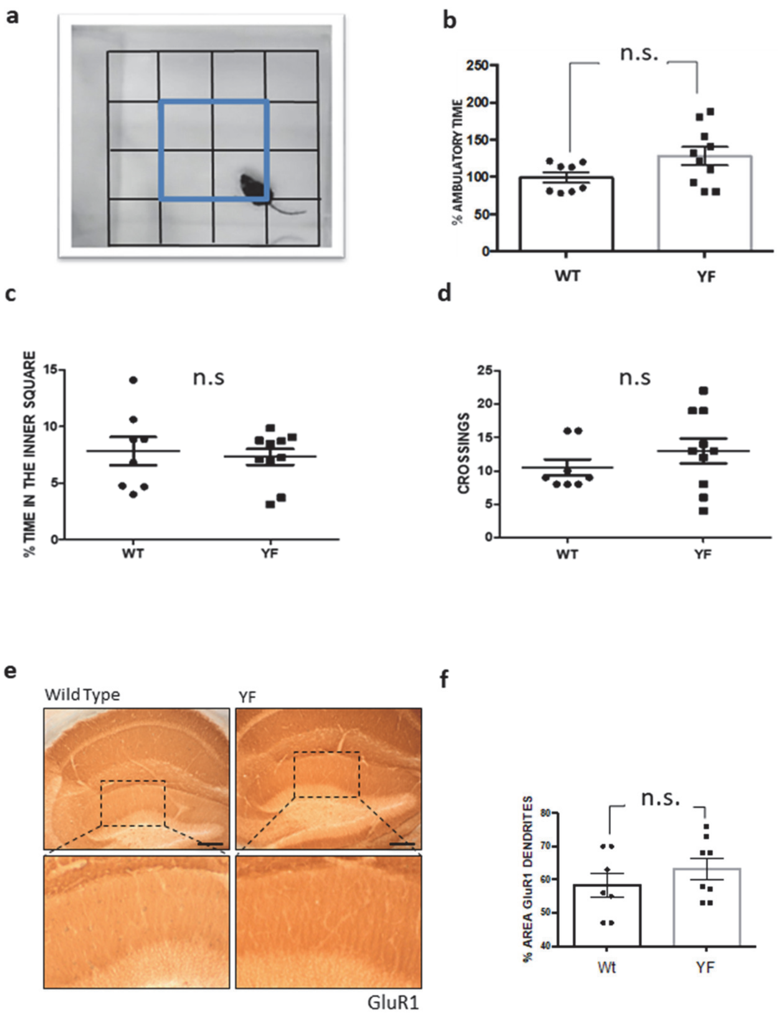

Figure S4. Effect of the expression of Yamanaka factors on parameters related to locomotion and anxiety using the open field test. (a) On the left a representative image of a mouse subjected to test. (b**)** To evaluate if the expression of YFs could modify some aspects related to mobility, the ambulatory time was carried out for wt and YFs-expressing mice. The ambulatory time represents the time in motion that the mice spend in the box for 10 minutes. The data is shown as a percentage of the average time spent, taking 100% the time spent by wt mice. No differences between WT and YFs-expressing mice were found although a non-significant increase was observed ($p = 0.084$). (c) To analyze the anxiety, the time that the WT and YFs-expressing mice enters in the inner square was registered. The data is shown as the percentage of the time the mice spend in the inner square. Crossings are also shown (d). No differences were observed in time spent in the inner square and crossings between the periphery and the center. Mean ± SEM; n.s.= non-statistically significant. **Effect of the cyclic induction of Yamanaka factors on GluR1 staining cells outside the granular layer.** (e) Representative images of GluR1-positive cells and their corresponding high-power magnifications in wild-type mice and YFs-expressing mice. (f) For quantifications, levels in the molecular layer. Dots: wild-type samples; Squares: YFs-expressing mice samples. Mean ± SEM; n.s.= non-statistically significant. Bars=200 µm.

Supplementary Experimental Procedure:

Open field test. Mice aged 10 months were used for this test (9–10 mice per group). The test was performed essentially as described previously (Hall, 1934; Seibenhener and Wooten, 2015), with some modifications. Firstly, the mice were deposited for 10 minutes on the corner of the 45x45 cm plastic opaque box with vertical walls and a grid drawn with sixteen identical squares on the ground, forming its four central squares the inner square. After each exposure, the cage was wiped with 70% ethanol to eliminate odors. Then, some parameters related to anxiety and locomotion were analyzed (ambulatory time, time in the inner square and crossings). For ambulatory time was recorded the time in motion that the mice spent in the box for 10 minutes. The data is shown as a percentage of the average time spent, taking 100% the time spent by WT mice. For the time in the inner square, the percentage of the total time that the mice entered the inner square with three legs was recorded. For the crosses, the times that the mice crossed the box through the inner square were quantified.

Bibliography:

Hall CS. (1934). Emotional behavior in the rat: defecation and urination as measures of individual differences in emotionality, J. Comp. Psychol. *18*, 385–403.

Seibenhener, M.L., and Wooten, M.C. (2015). Use of the Open Field Maze to measure locomotor and anxiety-like behavior in mice. Journal of visualized experiments : JoVE, e52434.
